# Correction: Price trends of reimbursed oncological drugs in Switzerland in 2005–2019: A descriptive analysis

**DOI:** 10.1371/journal.pone.0264610

**Published:** 2022-02-23

**Authors:** The PLOS ONE Staff

There is an error in the seventh sentence of the abstract. The correct sentence is: From 2005–2009 to 2015–2019, the median monthly product price over all distinct indications of all products decreased by 7.56% (CHF 5,699 [interquartile range 4,483–7,321] to CHF 5,268 [4,019–6,967]), whereas it increased by 73.7% for monoclonal antibodies.

The ORCID iD is missing for the second author. Author Christoph Jakob Ackermann’s ORCID iD is: 0000-0003-2621-5512 (https://orcid.org/0000-0003-2621-5512).

The publisher apologizes for the errors.
