# Supplementary material for: Price trends of reimbursed oncological drugs in Switzerland in 2005–2019: A descriptive analysis
Source: PLoS One. 2021 Nov 15;16(11):e0259936. doi: 10.1371/journal.pone.0259936 (PMC8592494; doi:10.1371/journal.pone.0259936)
Supplement: Attachment — Submitted filename: P2p_answers_21Sep2021.docx [file pone.0259936.s003.docx]

**Point-to-point answers to reviewers’ comments on PONE-D-21-18082R1**

Reviewer #1:

Unfortunately, I have the impression that except for the inflation effect being controlled by consumer price index, all other comments have been inadequately addressed or simply received a justification for leaving things as they are. So, following the non-compliance with adequate statistical analysis of essentially a "raw data set" and the extraction of conclusions associating price variations to public policy in the absence of any technical tool for reaching such a conclusion, I have to firmly propose for the paper to be rejected for publication.

Answer:

Our purely descriptive article presented hypotheses regarding possible effects of policy on prices, without claiming to prove causality. We realize that, despite explicitly calling our work “a descriptive analysis” in its very title, this was probably not understood by the reviewer. We cannot change the very nature of our article, but to avoid such misunderstandings by future readers, we now refrain from generating hypotheses regarding reasons for the observed price trends or of any other kind. Instead, our new discussion now addresses price differences between topographies and indication-specific pricing.

Just a remark on the issue of convergence: I insist convergence is a very well defined phenomenon in dynamic statistical analysis and the use of it under these circumstances would by itself suffice to have the paper rejected for non-compliance with the terms of PLOS One.

Answer:

In her/his first review, the reviewer had criticized that „convergence of prices is discussed as related to price decreases“, but in her/his second review, the issue changes from „what convergence is related to“ to the „definition of convergence“. This change of content of her/his critique makes it hard to understand what the reviewer was actually concerned with, and therefore hard to address her/his concerns.

While there is simply no such thing as „dynamic statistical analysis“, there are indeed mathematical fields like „dynamic data analysis“, or analysis of „dynamic models“ or „dynamical systems“ etc., which are about fitting differential equations to data (something totally unrelated to our article). In these disciplines, as in all of mathematics, convergence has indeed a technical, so called (ε, δ)-definition, and maybe that is what the reviewer meant. It is, though, most obvious that we never used the term „convergence“ in this sense, and we are convinced that no reader would assume that we meant it that way. We used the term in its colloquial and descriptive sense which we deem perfectly appropriate to describe the last facet of our figure 3. (Many words have very specific and well-defined meanings in specialized fields, but that constitutes no reason not to use them in their commonly understood sense in unrelated fields.) Moreover, wherever we used the word „convergence“ without direct reference to this figure 3, we have substituted the word, to avoid even the tiniest bit of confusion.

Reviewer #2:

I am happy with the revised version of the paper. I like the research question, the structure of the paper, the quality of writing, and the way the authors describe their empirical proceeding and results. Most importantly, the authors have addressed all the issues stated in my referee report for the first version appropriately.

Answer:

Thank you for this positive feedback.
