# Supplementary material for: Price trends of reimbursed oncological drugs in Switzerland in 2005–2019: A descriptive analysis
Source: PLoS One. 2021 Nov 15;16(11):e0259936. doi: 10.1371/journal.pone.0259936 (PMC8592494; doi:10.1371/journal.pone.0259936)
Supplement: Attachment — Submitted filename: Response to Reviewers - 27 Oct 2021.docx [file pone.0259936.s004.docx]

**Point-to-point answers to reviewers’ comments on PONE-D-21-30520**

**Reviewer #1:** This manuscript is well organized, and some issue should be revised before publication.

We would like to thank reviewer #1 for his positive opinion and suggestions on how to improve our article. We hope that our changes will meet your approval.

1. Consider shortening the conclusion in the abstract.

We removed what could be considered as part of the results section and focused on interpretation and implication of the results. Page 2, lines 52-54.

2. In the introduction, the fourth paragraph is too long. Would you revise it and focus on main goal?

Since the analysis is about drug price developments in Switzerland, we downweighed the comparison with the US and shortened the paragraph accordingly. The shortened paragraph now stresses the contrast of decreasing post-launch prices as observed in our current - and another recent – study with the overall situation of mostly rising launch and post-launch prices. We did not fully refrain from giving a very short overview over the literature since this was a request of a previous reviewer. Page 3, lines 84-87.

3. The figures should be edited to be more clear or, somewhat, attractive. Current ones look like the ones print screen from software without further editing.

We added pointwise “uncertainty bands” (± 1.96 standard errors) to all figures for improved clarity and visually improved all figures by removing the plain gray boxes and by using the more appealing Times font. We hope that this will mitigate the impression of print screens, and we will gladly implement additional “beautification” according to both reviewers’ specific suggestions!

**Reviewer #2:** This is a well-written manuscript that demonstrates a long-term exploration of the time trends in the price of anticancer drugs in Switzerland and attempts to find out what are the key cost drivers of the overall cost trend.

I believe that this study will help policies respond to the increasing cancer drug costs, and seek to identify whether these drugs are justified by a particularly high clinical benefit, which ultimately determines how best to systematically improve the underuse of high-value drugs and minimize the use of low-value drugs.

I have no issues to highlight in terms of content and statistical analysis. However, the figures and graphics in this manuscript are a bit messy for me. I think the author needs to think about how to make your artwork more clutter-free and visually appealing next time.

In short, I think this article is suitable for publication in PLOS ONE.

The authors would also like to thank reviewer #2 for appreciating our manuscript, in particular content and analysis. May we refer the reviewer to our above answer regarding figures and graphics? We would like to emphasize once again that we will gladly revise the figures further, according to specific requests.
